# Assessing Barriers to Uveitis Screening in Patients with Juvenile Idiopathic Arthritis Through Semi-Structured Interviews

**DOI:** 10.1097/pq9.0000000000000084

**Published:** 2018-06-13

**Authors:** Laura R. Ballenger, Stacy P. Ardoin, Kyla D. Driest

**Affiliations:** From the Division of Rheumatology, Nationwide Children’s Hospital, Columbus, Ohio.

## Abstract

**Introduction:**

Uveitis is a significant complication in patients with juvenile idiopathic arthritis (JIA) and can be asymptomatic until vision loss develops. Published guidelines recommend uveitis screening eye examinations every 3–12 months depending on multiple factors, but no literature evaluates adherence with and barriers to obtaining these screening eye examinations. This study assesses barriers in nonadherent patients to establish key drivers for future interventions.

**Methods:**

We identified patients with JIA who were nonadherent with uveitis screening guidelines through the electronic medical record (EMR). A rheumatologist conducted semistructured interviews with the patients or guardians regarding the patients’ most recent eye examinations, knowledge of the screening frequency, and barriers to completing the eye examinations. The results were qualitatively analyzed to determine any categorical variables present.

**Results:**

Ninety-two patients were identified as nonadherent, and the rheumatologist interviewed 45 patients or guardians. Categories identified following the interviews were system problems, access to care issues, and knowledge deficits. The largest category identified was system problems that included most recent eye examination not being in the EMR, the wrong eye provider identified in the EMR or difficulty with scheduling eye appointments.

**Conclusions:**

This qualitative study identified categories of barriers to obtaining screening eye examinations in patients with JIA. Identification of these barriers will facilitate the development of a specific aim and key driver diagram to guide future quality improvement interventions.

## Abstract 2

Supplemental Digital Content is available in the text.

## INTRODUCTION

Juvenile idiopathic arthritis (JIA) is a form of childhood arthritis that begins before 16 years of age and is of unknown etiology.^1^ The estimated worldwide prevalence is around 16–35 per 100,000 children with higher prevalence in North America and White children.^2^ The International League of Associations of Rheumatology classifies patients with JIA into 6 subtypes.^1^

JIA is a commonly identified cause of uveitis in children in the United States.^3^ Uveitis is a complication in 10–30% of patients with JIA^4^ and is more common in patients with the oligoarticular subtype of JIA, younger age at diagnosis, a positive antinuclear antibody, a negative rheumatoid factor, and a negative anticyclic citrullinated peptide antibody.^5^

Children with JIA-associated uveitis are at risk to have a severe, chronic, insidious course of their uveitis.^6^ The first episode of uveitis is more common with active arthritis; however, relapses more commonly present in the absence of arthritis.^7^

Symptoms of uveitis may include redness, pain, photophobia, and blurry vision, but uveitis associated with JIA can be asymptomatic.^8^ Patients with JIA-associated uveitis have a higher rate of complications than patients with uveitis from other causes,^6^ including cataracts, glaucoma, ocular hypertension, band keratopathy, posterior synechiae, retinal detachment, severe visual impairments, and blindness.^4,9^ Some patients require surgical procedures for these complications, including cataract extraction.^4^

Current guidelines recommend that patients with JIA have regular uveitis screening examinations with an ophthalmologist. The current recommendations for uveitis screening in patients with JIA are based upon a population-based study from Germany by the Heiligenhaus group. Screening frequency is dependent upon patient age at diagnosis, time since diagnosis, type of JIA, and antinuclear antibody (ANA) status. Patients may require screening examinations as frequently as every 3 months based on the guidelines.^10^ Table 1 shows the screening guidelines utilized within the Nationwide Children’s Hospital (NCH) Rheumatology Clinic based upon the Heiligenhaus group’s recommendations.

**Table 1.**
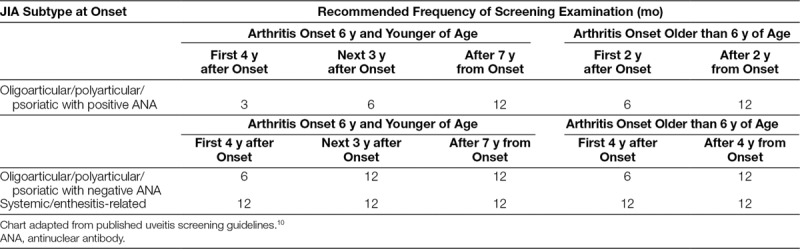
NCH Rheumatology Clinic Uveitis Screening Recommendations

To the best of our knowledge, no published literature addresses patient adherence with eye screening recommendations in JIA. Before initiation of this study, about 70% of all patients with JIA in the NCH Rheumatology Clinic were adherent with uveitis screening guidelines outlined in Table 1. The goal of this study was to assess barriers to obtaining screening eye examinations in patients with JIA to establish a specific aim and key drivers for future quality improvement efforts to improve adherence with the eye screening examinations.

## METHODS

This qualitative study was performed at NCH in Columbus, Ohio, within the Rheumatology Clinic. The study was not human subjects research based upon institutional guidelines, and the institutional review board did not require prior approval. Inclusion criteria were patients with JIA by International Classification of Disease Ninth Edition (ICD-9) codes, a follow-up appointment scheduled within 2-months, nonadherence to the screening guidelines as determined by documentation of the most recent eye examination report in the electronic medical record (EMR). Patients had to be English-speaking to participate in the study.

One of the rheumatologists contacted the legal guardians of the patient (or the patient if over 18 years of age) by phone about 1 week before the scheduled follow-up appointment. One phone call attempt was made to each phone number listed within the EMR including home, mobile, and work phones.

The rheumatologist conducted a semistructured interview with both open- and closed-ended questions (see Appendix, available as **Supplemental Digital Content** at http://links.lww.com/PQ9/A28, for interview questions). The interviewer took hand written notes with direct quotes and additional field notes about the interviewer’s observations and thoughts following the interview. We coded the responses to each question as either positive or negative for barriers. The interviewer reviewed the notes and coding twice to create categorical variables from the identified barriers. We determined the relative importance of each of the variables from the percentage of patients identifying each barrier.

Additionally, we reviewed each patient’s chart for a uveitis letter. The uveitis letter is a document developed by the NCH Rheumatology Clinic as a method for communicating with eye providers regarding the risk of uveitis in JIA and the recommended eye screening examination frequency. At the time of JIA diagnosis, the rheumatology provider generates the letter within the EMR, provides a copy to the patient and instructs the patient to share the letter with their eye providers.

## RESULTS

Over the 2-month duration of the study, we identified 246 patients with JIA with upcoming appointments in the Rheumatology Clinic. Ninety-two (37.4%) of these patients were nonadherent with their uveitis screening examinations based on review of the EMR. Forty-five (49% of the nonadherent patients) patients or their guardians completed interviews. The other 47 families could not be reached by phone to complete an interview. Patient ages ranged from 3 to 22 years. The majority of the interviewees were the mothers of the patients.

Review of the interview notes identified barrier categories of (1) system problems; (2) knowledge deficits; and (3) access to care issues. Some interviewees identified more than 1 barrier. Table 2 shows the barrier categories, specific barriers included in each category, and specific comments from interviewees within each category.

**Table 2.**
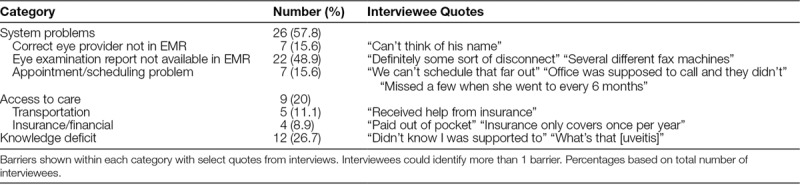
Interview Results by Category

System problems accounted for the most substantial proportion of barriers with 57.8% of patients identifying at least 1 system problem. Almost half (48.9%) of the nonadherent patients were up-to-date based on the date of most recent eye examination reported by the interviewee. Comments from interviewees included “I’m on top of this” and “definitely some form of disconnect” between the rheumatology clinic and the eye providers. Seven patients (15.6%) had incorrect documentation of their eye provider in the EMR, which is problematic because faxed requests for eye examinations are sent to the eye provider listed in the EMR. Seven patients (15.6%) identified difficulty with scheduling appointments with their eye providers as a barrier. Many reported that the eye provider appointments were not available beyond a 3-month time frame. Some stated that it was easier to schedule appointments when examinations were needed every 3 months rather than every 6 months or beyond because they could schedule a follow-up visit at the end of their eye appointment. Comments from interviewees included “we honestly just forgot… we can’t schedule out that far” and “missed a few when she went to every 6 months.”

Knowledge deficit was the next most identified barrier with 26.7% of interviewees stating they were unaware of what uveitis was or how often screening examination should be performed. Seven of the interviewed patients (15.6%) did not have a uveitis letter documented in the EMR although not all of these patients had an identified knowledge deficit. Fifty-one of the 246 patients identified as nonadherent for inclusion in this study (20.7%) did not have a uveitis letter documented in the EMR.

Access to care was the least cited barrier with 9 interviewees (20%) identifying problems related to transportation (5 interviewees, 11.1%) or finances (4 interviewees, 8.9%). Most families that identified transportation barriers as an issue utilized transportation resources such as those provided by their insurance company. Only 1 parent was unaware of possible resources, and this person was unable to drive long distances due to a health condition. Most of the financial barriers identified related to the frequency of screening eye examinations allowed by the vision insurer. Some parents were not aware that eye examinations for JIA could be billed under their medical insurance.

## DISCUSSION

Our study identified multiple potential barriers to obtaining screening eye examinations in the patient population with JIA. We categorized the barriers as system problems, knowledge deficits, and access to care issues. We expected that access to care barriers would be more common for families, but these were the least cited barriers. A large proportion of the nonadherence with screening examinations seems related to lack of available documentation of the most recent eye screening examination.

**Fig. 1.**
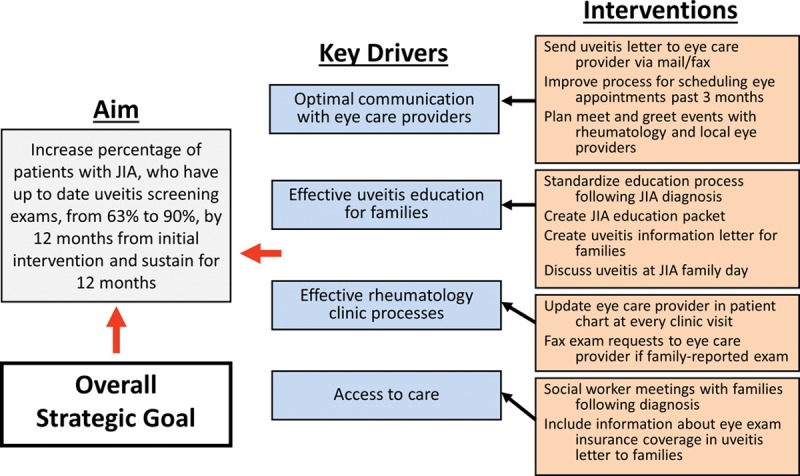
Proposed specific aim and key driver diagram with possible future interventions.

Identification of these barriers helped our team to establish key drivers for future quality improvement interventions. The proposed key driver diagram based on our institutional templates is shown in Figure 1. Baseline adherence data are quoted from this study. Key drivers derived from this project are optimal communication with eye providers, effective rheumatology clinic processes, effective uveitis education for families, and access to transportation. Multiple potential future interventions are suggested in the figure and will be implemented moving forward from this project in our rheumatology clinic.

Several limitations exist in the study. First, we did not confirm if interviewees provided accurate information of most recent eye examinations by retrieving eye provider records. System problems were the most frequently identified barrier with the lack of documentation of an up-to-date eye examination reported by the family common. Social desirability might have been present if patients were telling the interviewer answers that they perceived the interviewer wanted to hear. Second, patients had to have a future appointment scheduled to be included in the study. However, many patients may not have scheduled their rheumatology follow-up appointments. If patients are nonadherent with rheumatology follow-up visits, they may also be nonadherent with their uveitis screening examinations. Third, selection bias was also present as only families with working telephone numbers that were English-speaking could participate in the project. Working or single parent families may not have been able to answer the phone. Lastly, interviewer bias may be a limitation of this single-reviewer qualitative study.

## CONCLUDING SUMMARY

In conclusion, our study identified common barriers to obtaining screening eye examinations in patients with JIA. Clinicians caring for patients with JIA should consider possible barriers to obtaining uveitis screening examinations in their patient population. It is crucial for them to understand these barriers before making interventions to increase adherence to screening guidelines.

## Supplementary Material
