# Supplementary material for: Assessing Barriers to Uveitis Screening in Patients with Juvenile Idiopathic Arthritis Through Semi-Structured Interviews
Source: Pediatr Qual Saf. 2018 Jun 13;3(3):e084. doi: 10.1097/pq9.0000000000000084 (PMC6132814; doi:10.1097/pq9.0000000000000084)
Supplement: Supplementary file 1 [file pqs-3-e084-s001.docx]

Appendix: Interview Questions

Who is your current eye care provider?

When was your child’s last eye exam?

Can you tell me how often your child’s eye exams should be performed?

What difficulties, if any, have you had getting an appointment to see your eye care provider?

Have you had any financial or insurance concerns associated with your child’s eye exams?

Have you had any difficulty with transportation to your child’s eye care provider?

What other barriers have your encountered in obtaining screening exams for uveitis?

Do you have any other comments or concerns regarding screening your child for uveitis?
